# Very severe COVID-19 in the critically ill in Tunisia

**DOI:** 10.11604/pamj.supp.2020.35.136.24753

**Published:** 2020-08-06

**Authors:** Imen Ben Saida, Emna Ennouri, Rayane Nachi, Khaoula Meddeb, Jihene Mahmoud, Nesrine Thabet, Salma Jerbi, Mohamed Boussarsar

**Affiliations:** 1 Medical Intensive Care Unit, Farhat Hached University Hospital, Faculty of medicine, University of Sousse, 4000, Sousse, Tunisia,; 2 Research Laboratory N° LR12SP09, Heart Failure, Farhat Hached University Hospital, 4000, Sousse, Tunisia

**Keywords:** SARS-CoV-2, acute respiratory distress syndrome, pneumonia, COVID-19

## Abstract

**Introduction:**

SARS-CoV-2 is an emerging health threat outbreak. It may cause severe viral pneumonia with Acute Respiratory Distress Syndrome requiring critical care. Aim: to describe clinical features and outcomes of critically ill patients with SARS-CoV-2 infection.

**Methods:**

it was a retrospective study carried out in the medical ICU of Farhat Hached teaching hospital between March 11 and May 7, 2020. All consecutive patients with RT-PCR confirmed COVID-19 were included. Clinical characteristics and outcomes were collected by reviewing medical records.

**Results:**

during the study period, 10 critically ill patients with COVID-19 were enrolled. Mean age, 51.8±6.3 years; 8(80%), male. The most common comorbidities were; diabetes mellitus, 6(60%), obesity 2(20%), chronic kidney disease 2(20%) and hypertension 1(10%). Mean SAPS II, 23.2±1.8. The mean arterial oxygen partial pressure to fractional inspired oxygen ratio at admission was 136.2±79.7. Noninvasive mechanical ventilation was used in 4(40%) patients and 7(70%) received invasive mechanical ventilation. Tidal volume and PEEP were set respectively within the median [IQR] of, 5.7[5.6-6.3]ml/Kg and 10.7[6.5-11.7]cm H_2_O. Plateau pressure was monitored in the median [IQR] of 27.9 [25.9-28.5] cm H_2_O. Four patients received hydroxychloroquine alone and five hydroxychloroquine associated with an antiviral. Five patients developed respectively hyperactive (n=2), hypoactive (n=2) and mixed delirium (n=1). Mortality rate was at 70%.

**Conclusion:**

this study demonstrated a particular profile of COVID-19 in the critically ill as a severe presentation in aged males with comorbidities presenting with an ARDS-like and neurological impairment with poor prognosis. The only survivals seem to have benefited from noninvasive ventilatory support.

## Introduction

Severe acute respiratory syndrome coronavirus-2 (SARS-CoV-2), the novel corona virus first detected in Wuhan, China on December 2019 is the pathogen causing coronavirus disease 2019 (COVID-19) [1]. It is a worldwide public health emergency. The outbreak was declared by the World Health Organization (WHO) as pandemic on March 11, 2020 [2]. The first confirmed case in Tunisia was reported on March 3, 2020 [3]. The detection of this case has led to the implementation of high-level preventive strategies, already planned since February 2020, including physical distancing measures by Tunisian government. The COVID-19 may cause severe viral pneumonia with Acute Respiratory Distress Syndrome (ARDS) requiring critical care. Information about Tunisian critically ill patients with COVID-19 is scarce. To the best of the authors´ knowledge, this is the first report of the clinical features and outcomes of critically ill patients with COVID-19 in Tunisia. The aim of the present study was to describe the demographic characteristics, clinical presentation, imaging findings, management strategies and challenges, and outcomes of critically ill patients with SARS-CoV-2 infection.

## Methods

**Study design and participants:** it is a retrospective study carried out in a 9-bed medical intensive care unit (ICU) of Farhat Hached teaching hospital (Sousse, Tunisia) between March 11, 2020 and May 7, 2020. All consecutive patients with confirmed COVID-19 infection were included. A confirmed case of COVID-19 is defined by an RT-PCR positive result testing of a specimen collected on a nasopharyngeal swab or endotracheal aspirate sampling in intubated patients. There were no non-inclusion criteria.

**Data collection:** data were collected by reviewing the medical records. The following patients´ demographic and clinical characteristics were collected: age, gender, past medical history, Charlson comorbidity index (CCI) [4], the Simplified Acute Physiology Score (SAPSII) [5], timeline between the illness onset to ICU admission, clinical symptoms or signs at presentation, exposure history, laboratory and radiologic results, management strategies (i.e., antiviral therapy, antibiotics, vasopressor, corticosteroid therapy, kidney replacement therapy, ventilatory support, respiratory indices of mechanical ventilation including the ratio of Partial Pressure of Arterial Oxygen and Fraction of Inspired Oxygen (PaO2/FIO2 ratio), Tidal volumes/ Predicted Body Weight (PBW), plateau pressure (Pplat) and Positive End-Expiratory Pressure (PEEP)), delirium assessed by the Tunisian version of the confusion assessment method (CAM-ICU) [6] and outcomes including length of stay and mortality.

**Definitions:** COVID-19 was diagnosed based on the criteria published by the WHO and confirmed by RT-PCR assay of specimens obtained by nasopharyngeal swab or endotracheal aspirate [7]. SAPSII Score is a severity score and mortality estimation tool and made of 12 physiological variables and 3 disease-related variables. The worst physiological variables are collected within the first 24 hours of ICU admission. CCI is a weighted index that takes into account the number and the seriousness of comorbid disease to estimate the risk of death from comorbid conditions. It is used as a measure of comorbidity burden. Acute Respiratory Distress Syndrome (ARDS) was diagnosed according to the Berlin Definition [8]. Confusion Assessment Method (CAM-ICU) [6] is the most widely used tool for delirium assessment in ICUs. Richmond Agitation-Sedation Scale [9]: It is a scale used to evaluate the level of alertness or agitation of patients under sedatives.

**Statistical analysis:** statistical analyses were performed with SPSS software. The Shapiro-Wilk test was used to verify the normality of distribution of continuous variables. Descriptive statistics were computed for all study. Categorical data were presented as numbers (%) and continuous ones as mean ± standard deviation or as median (interquartile range 25-75), as appropriate.

## Results

**Demographic and clinical characteristics:** during the study period, 10 critically ill patients with SARS-CoV-2 infections were enrolled, out of 37 patients admitted in the same period for a suspected COVID-19 clinical presentation. Patients´ demographic and clinical characteristics are shown in Table 1. The mean age was 51.8±6.3years. Eight (80%) were male. Mean SAPS II was 23.2±1.8. The mean CCI was 2.8±0.4. The most common comorbidities were diabetes mellitus (60%), obesity (20%), chronic kidney disease (20%) and hypertension (10%). The most common initial symptoms were fever, shortness of breath and cough. All the patients were admitted in the ICU for hypoxemic acute respiratory failure. Seven (70%) patients were referred from infectious diseases ward.

**Table 1:** COVID-19 patients' demographics and characteristics at ICU admission

	Study population n=10
**Age** (years), Mean±SD	51.8±6.3
**Male**, n (%)	8(80)
**Comorbidities**, n(%)	
Hypertension	1(10)
Diabetes	6(60)
Obesity	2 (20)
Asthma	1(10)
Chronic kidney disease	2(20)
**CCI score**, Mean±SD	2.8±0.4
**SAPS II**, Mean±SD	23.2±1.8
**History of travel and contacts**, n(%)	
History of travel to country where Covid-19 is endemic	1(10)
Known sick contact	2(20)
**Mean duration of symptoms before ICU admission**	
(days), Mean±SD	11.3±1.2
**Initial Symptoms**, n(%)	
Shortness of breath	9(90)
Sore throat	6(60)
Cough	8(80)
Headache	2(20)
Rhinorrhea	6(60)
Fever	10(100)
**Laboratory data on admission**	
White-Cell Count (elt/mm3), Mean±SD	12535±366.2
Lymphocytopenia, n(%)	8(90)
Creatinine <110 µmol/l, n(%)	8(80%)
**Imaging**, n(%)	
Chest X-Ray	7(70)
Chest CT scan	5(50)
**Chest X-Ray findings**, n(%)	
Clear	0/7(0)
Bilateral infiltrates	4/7(40)
Pleural effusion	1/7(20)
Atelectasis	6/7(50)
**Chest CT findings**, n(%)	
Bilateral ground-glass opacification	4/5(80)
Patchy consolidation	1/5(20)
Nodules	1/5(20)
Pleural effusions	1/5(20)
**ARDS**, n(%)	
Mild	2(20)
Moderate	4(40)
Severe	4(40)
**PaO2/FIO2 ratio**, Mean±SD	136.2±79.7

CCI, Charlson comorbidity index; Countries with endemic Covid-19 disease included China, Iran, Italy, France, Egypt, Turkey, Spain and united states; Obesity is defined by a body mass index ≥30kg/m2; CT, Computed tomography; ARDS, Acute Respiratory Distress Syndrome; PaO2/FiO2, arterial partial pressure of oxygen by fraction of inspired oxygen ratio.

**Laboratory and radiologic findings:** the first test for COVID-19 was positive in 9 patients out of 10. Only one patient had a negative first test and positive repeat test. Laboratory and radiological findings are summarized in Table 1. Lymphocytopenia occurred in 8(80%) patients. A chest radiograph was done for 7 patients. A computed tomography (CT) scan of the chest was done for five (50%) patients. The common chest CT findings were ground-glass opacities in 4(80%) patients and patchy consolidations in one patient (20%).

### Treatment and outcomes

**Figure 1:**
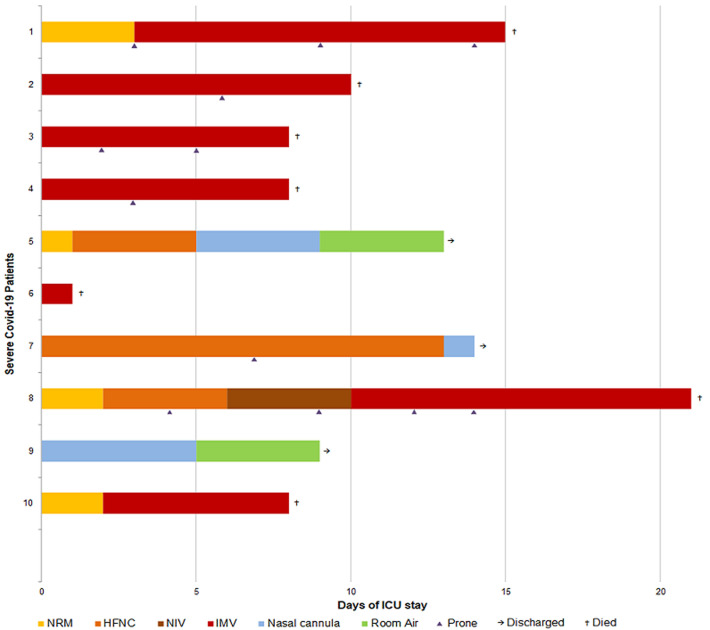
ventilatory support, prone positioning and outcomes for COVID-19 patients included in the study

**Figure 2:**
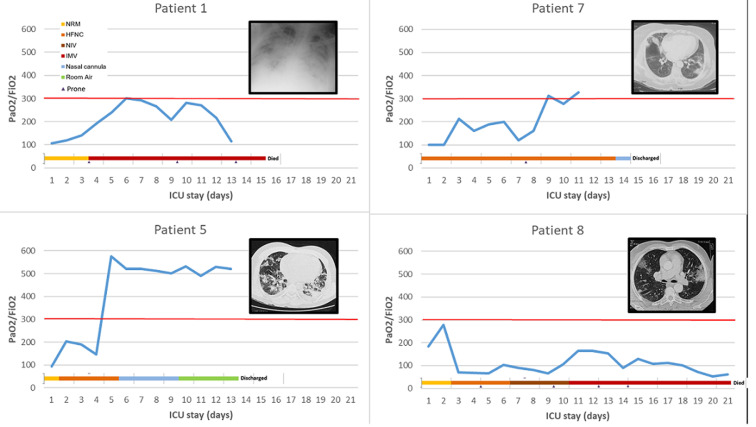
daily dynamical changes of PaO_2_/FiO_2_, according to respective ventilatory supports in four different profiles initially non-invasively managed, in COVID-19 patients 1, 5, 7 and 8

*Ventilatory management:* Figure 1 describes patients´ ventilatory support during their ICU stay. While the four initial patients were rapidly even immediately intubated, the five secondarily admitted were managed by different non-invasive devices including non-rebreathing mask (NRM), High-Flow Nasal Cannula (HFNC) and Non-Invasive Ventilation (NIV). This shift in the management was motivated by the worse outcome of the initial invasively managed patients. Figure 2 illustrates gradual improvement of PaO_2_/FiO_2_ in case 5 under HFNC doubled by NRM to reduce aerosolization, albeit poor initial PaO_2_/FiO_2_. This improvement was significantly increased after a session of prone positioning (6-hour long session) at day 9, while this patient was presenting diffuse alveolar consolidation at his second CT scan performed at ICU admission. Another patient (case 8) managed non-invasively was rather unresponsive to a myriad of ventilatory procedure as NRM, HFNC and NIV. The invasive mechanical ventilation (IMV) was mandatory at day 10.

**Figure 3:**
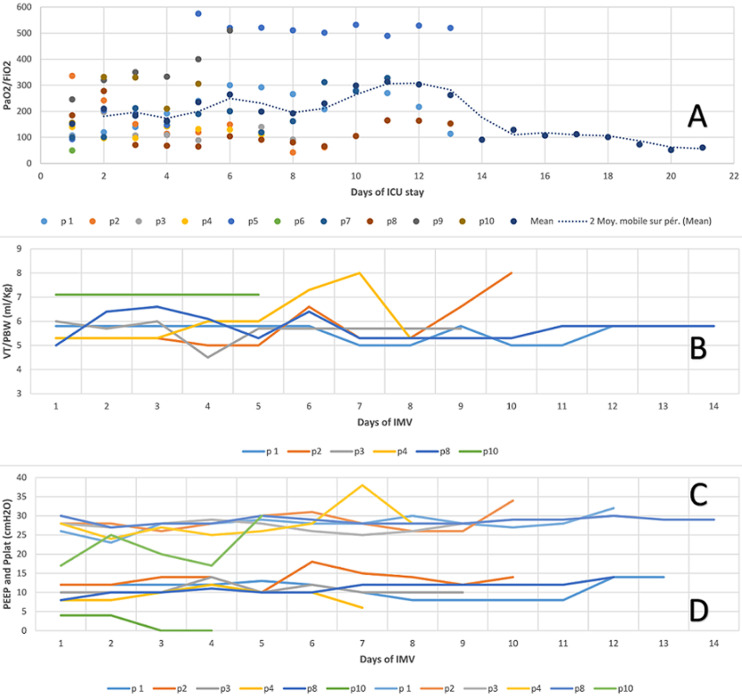
daily dynamical changes in respiratory indices of mechanical ventilation in patients with COVID-19 included in the study

As shown in Table 2, seven (70%) patients required IMV with six of them needing prone positioning (18-hour long sessions). For patients with IMV, ventilatory settings were based on ARDS management strategies. The mean PaO_2_/FiO_2_at admission day was 136.2±79.7. Surprisingly, all severe patients immediately intubated upon arrival, suffered severe but well-tolerated hypoxemia. Daily means PaO_2_/FiO_2_were less than 300 and frequently less than 150, consistent with moderate-to-severe ARDS (Figure 3). Figure 3 shows daily dynamical changes in respiratory indices of mechanical ventilation parameters. Tidal volume/ Predicted Body Weight set by the attending physicians ranged between 4.5 and 8ml/kgPBW with a certain variation between and intra-patients. PEEP was set between 0 and 18cm H_2_O. Plateau pressure was often monitored below 30cm H_2_O, as recommended by protective ventilation. As a result of these settings, Tidal volume and PEEP were set respectively within the median [IQR] of, 5.7[5.6-6.3]ml/KgPBW and 10.7[6.5-11.7]cm H_2_O. Plateau pressure was monitored in the median [IQR] of 27.9[25.9-28.5] cm H_2_O.

**Table 2:** COVID-19 patients' therapeutic characteristics and outcomes

	Study population; n=10
**Ventilatory support**, n(%)	
HFNC	3(30)
NIV	1(10)
IMV	7(70)
**Prone position**, n(%)	
HFNC	2(20)
IMV	5(50)
**Neuromuscular blockade**, n(%)	7(70)
**Hydroxychloroquine**, n(%)	9(90)
**Lopinavir/Ritonavir**, n(%)	4(40)
**Oseltamivir**, n(%)	1(10)
**Ruxolitinib**, n(%)	1(10)
**Antibiotics**, n(%)	10(100)
**Vasoactive drugs**, n(%)	7(70)
**Delirium**, n(%)	5/7 (71.4)
**Length of stay**, (days) Mean±SD	11.2±5.8
**Mortality**, n(%)	7(70)

HFNC, High-Flow Nasal Cannula; NIV, Noninvasive Ventilation; IMV, Invasive Mechanical Ventilation

*Pharmacological treatment:* six patients received sedatives agents and neuromuscular blockade. Seven (70%) received vasoactive drugs. Two patients received renal replacement therapy, one for an acute renal failure consecutive to acute tubular necrosis (case 10) and one for a chronic renal failure on peritoneal dialysis (case 7). Treatment options of patients are presented in Table 2. Nine patients received hydroxychloroquine among them 5 patients also received antivirals (lopinavir/ritonavir or oseltamivir). Antibiotic were used in all patients: 5 patients received cefotaxime and ofloxacin and the others received azithromycin. Only one patient received ruxolitinib. Systemic cortocosteroids was used only in one patient for asthma exacerbation. Curative anticoagulation was introduced in 8 patients. No patient received intravenous immunoglobulin. None of the severe patients received extracorporeal membrane oxygenation in the present study. Based on sudden-onset severe hypoxemia, clinical signs, radiological and echocardiographic findings, empirical thrombolysis was done for three hemodynamically unstable patients highly suggesting pulmonary embolism.

### Outcomes

*Neurological impairment:* sustained polyuria, important and well-tolerated fever without evidence of infection and large blood pressure variation suggesting vegetative disorders were noticed in four patients. During the ICU stay, 7 patients out of 10 (those having a Richmond Agitation-Sedation Scale greater than or equal to “-3”) were assessed for delirium. Five patients out of 7 developed respectively hyperactive (n=2), hypoactive (n=2) and mixed delirium (n=1).

*ICU course and mortality:* the mean ICU length of stay was 11.2±5.8 days. Of the 10 patients, 7(70%) had died and 3 had been discharged from the ICU. Causes of death were sudden refractory hypoxemia (4/6, 50%), septic shock secondary to a probable ventilator associated pneumonia (case 3) and refractory hypovolemic shock with acute kidney injury and tubular necrosis (case 10). One patient, admitted with a gasping respiration, died within the first hour albeit immediate appropriate management (case 6). Biphasic evolution was noticed in some patients (cases 1, 2 and 3). Sudden refractory hypoxemia occurred often after a significant stabilization period. In the first patient this happened within the weaning process after achieving a P/F near 300 for 7 days.

## Discussion

This study describes 10 critically ill patients with COVID-19 admitted for acute hypoxemic respiratory failure. The main findings of the present retrospective study were: i) Most patients were older male with chronic underlying conditions. ii) All patients were admitted to the ICU because of acute hypoxemic respiratory failure and most of them needed endotracheal intubation and invasive mechanical ventilation. Three patients were completely managed with noninvasive mechanical ventilation. iii) The mortality rate was at 70%. The present study had two limitations. First, it was a retrospective study conducted in a single center. Second, the small number of patients, only patients admitted in ICU were included. Thus, future studies with larger sample sizes and prospective study design are needed to better describe the profile and the outcomes of critically ill patients with COVID-19. However, to the best of authors´ knowledge, this is the first report of critically ill patients admitted for SARS-CoV-2 in Tunisia. As it was in previous reports [1,2,10,11], COVID-19 affected older male patients with comorbidities. Similar to previous investigations [1,12,13], patients with underlying medical conditions most commonly diabetes, obesity and chronic kidney disease were at higher risk for severe illnesses. The patients in the present study had similar symptoms to those described in reports from china, Italy and United States [1,2,14]. Fever, shortness of breath and cough were present in almost all patients. In line with previous reports [2], ARDS and refractory hypoxemia were the main reasons for ICU admission. Similar to other previous reports [1,14], lymphocytopenia was common.

In the present study, 30% of patients were completely managed with noninvasive mechanical ventilation, whereas 70% required endotracheal intubation. The use of IMV in the present study was similar to the report by Arentz *et al*. (Washington states) [15]. However, this rate was lower compared with the data reported by Grasselli et al [2] in an Italian ICU but higher than other reports from Wuhan, China in which the need for endotracheal tube varied from 15% to 47% [10,14,16,17]. This discrepancy in the rates of ventilatory support may be explained by different severity of hypoxemia (PaO_2_/FiO_2_) and differences in thresholds for ICU admission between studies. Altered gas exchanges was detected in most patients. The mean PaO_2_/FiO_2_in this study was 136.2±79.7. Thus, ventilatory settings were based on ARDS management strategies including low tidal volumes and PEEP titration. The uniformity of hypoxemia in enrolled patients contrasted with a spectrum of different respiratory mechanics attested by patients reported respiratory pressures and responsiveness to prone position. This variability was described by Gattinoni [18], who proposed different phenotypes of respiratory distress in COVID-19 illness according to radiological findings and respiratory mechanics and suggested different ventilatory management strategies for each phenotype. It is important to notice that the variability in visco-elastic respiratory system properties between patients, was also detected in a same patient during his ICU course.

Regarding pharmacological treatment, different associations of antimicrobial agents were administered. Four patients received Lopinavir/Ritonavir; one patient received Oseltamivir and the later patient received Ruxolitinib. Those drugs were used empirically without proof of their efficacy [1]. Nine patients out of ten received hydroxychloroquine in the present study. Expert from China [19] and from Italy [20] recommended the use of chloroquine or hydroxyl-chloroquine in COVID-19 patients, given a potential role in clinical success and outcomes improvement [19,21]. However, more evidence-based data is still required. The heterogeneity in the therapeutic strategy could be explained by daily emerging data on COVID-19 pathophysiology and therapeutic options and by the lack of consensus [22]. In fact, the first therapeutic strategies were extrapolated from existing clinical data derived from other viruses including SARS-CoV-1, Middle East respiratory syndrome coronavirus, and non-coronaviruses (eg, Ebola virus disease). All patients in the present study initially received antibiotics. It has been suggested that like seasonal influenza, COVID-19 infection may be associated with bacterial coinfection. Only one patient received glucocorticoids for asthma exacerbation. There are conflicting positions regarding corticoids in patients with COVID-19 [1,23]. Further studies are needed to determine the benefit or not of systemic glucocorticoids in those patients.

Curative anticoagulation was introduced for eight patients in the present study. In fact, some authors [10,24,25] reported high D-dimers concentrations and an increased coagulation activity in patients with COVID-19 pneumonia and that it is associated with fatal outcome. The biphasic evolution of some patients can be explained by an excessive inflammatory response with cytokine storm causing extensive lung damage and occurring within the first week in invasively ventilated patients in this study (cases 1, 2 and 3) [17,26]. Delirium was observed in 5 out of 7 COVID-19 patients screened by the Tunisian version of the CAM-ICU, while the previous reported incidence in non COVID-19 period, was around 36% [6]. The important increase of delirium rate may be explained by supplemental factors related to the specificity of the SARS-CoV2 itself. A potential direct action [27], or an indirect one via inflammatory mediators on central nervous system is possible [28]. The important consequent social changes, in addition to typical deliriogenic factors omnipresent in the ICU such as sedatives, prolonged mechanical ventilation and immobility may have played a role. In fact, measures of social distancing may be a contributory risk factor for delirium in older adults, who have less or no family visitation and limited mental and spiritual support from caregivers [29]. It is important to notice also that elderly patients, who are at greatest risk to develop severe COVID-19 forms, are also those who usually develop delirium in ICU.

The mortality rate in the current study was at 70%. It is in line with previous reports in which the mortality rate varied between 16% and 78% [10,16,17,26]. The main cause of death was refractory hypoxemia. Albeit, ARDS itself could explain severe hypoxemia, pulmonary embolism was also discussed but never confirmed in this study because of the high severity of the presentation impeding transport to perform CT Angiography of the chest. Previous studies have reported a high incidence of thrombotic complications in COVID-19 illness, reaching 30% [30-32].

## Conclusion

The present study is very peculiar by the very severe presentation of the initial patients that exhibited an ARDS like presentation associated with a neurological impairment but surprisingly without acute renal failure. The non-invasive early management achieved better prognosis.

### What is known about this topic

- The Covid-19 was declared by the World Health Organization (WHO) as pandemic on March 11, 2020;
- Data on patient’s characteristics, clinical presentation, imaging findings, management strategies, and outcomes of critically ill patients with SARS-CoV-2 infection are heterogeneous between different studies and countries.

### What this study adds

- Finding demonstrated a peculiar profile of COVID-19 in the critically ill as a severe presentation in aged males with comorbidities and poor prognosis;
- Most patients have an ARDS-like presentation and neurological impairment but no acute kidney injury;
- The only survivals seem to have benefited from noninvasive ventilatory support.
